# Increase in breeding bird abundance and diversity with semi-natural habitat in vineyard landscapes

**DOI:** 10.1371/journal.pone.0284254

**Published:** 2023-08-21

**Authors:** Verena Rösch, Gina Hafner, Jo Marie Reiff, Martin H. Entling

**Affiliations:** iES Landau, Institute for Environmental Sciences, RPTU Kaiserslautern-Landau, Kaiserslautern, Germany; Feroze Gandhi Degree College, INDIA

## Abstract

Agricultural expansion and intensification are major threats to biodiversity, and even some once common farmland bird species are now endangered. Wine-growing landscapes are intensively managed but can still be an attractive habitat for a wide range of species. However, only few bird species breed within vineyards and thus, semi-natural habitat types like hedges, woodland patches and grasslands are crucial for bird populations. We investigated how birds breeding in wine-growing areas are influenced by the surrounding landscape at three spatial scales: territories, sampling transects and landscapes. In the German wine growing region Palatinate, sixteen landscapes with a radius of 500 m were chosen spanning a gradient in the cover of semi-natural habitat. Bird territories were mapped along three transects of 500 m length in each landscape. We found 300 territories of 33 bird species. Positive effects of semi-natural habitat cover on birds were strongest at the transect scale, with almost proportional increase of species and territory numbers with the cover of semi-natural habitat. Most bird species selected territories that contained more semi-natural habitat than the landscape-wide average of 13.5%, but e.g. woodlark and linnet showed an opposite preference. In addition, the birds’ community composition was influenced by the composition of the surrounding landscape. Most species were associated with semi-natural habitat types or built-up areas while vineyards had hardly any species associated with them. Our results suggest that in wine-growing landscapes, the decline in farmland birds can be reversed by the re-establishment of hedges, trees, woodland patches, traditional orchards and grassland areas. However, as preferences at the territory scale were species-specific, there is no uniform best solution for bird conservation in viticultural landscapes. Thus, landscape development should always be accompanied by experts that take the demands of existing and potential breeding birds into account.

## Introduction

Biodiversity associated with agricultural habitats is declining at an alarming rate [1, 2] and the losses in arthropod biomass over recent decades [3–5] are passed on to subsequent trophic levels. Accordingly, the populations of many bird species that occur in agriculturally used areas are declining [6–9] and in many parts of Europe even once common farmland bird species such as barn swallow, yellowhammer and starling are now increasingly threatened [8, 10–12].The main drivers of biodiversity loss are changes in management practices including an increase in the use of agrochemicals and fertilisers, landscape simplification through larger field sizes and the removal of semi-natural habitat (SNH) elements like hedges, woodland patches, tree lines, permanent grasslands and fallows [13–15]. While the area of SNH types is often small, their contribution to the landscape-wide biodiversity is considerable [16]. The diversity of various groups of invertebrates have been shown to depend on the wider landscape surrounding their habitat [17–20]. For birds too, the composition of the landscape has been shown to affect their species richness, number of territories and community composition both during the breeding season and in winter [21, 22]. However, the spatial scale of dependence has rarely been studied explicitly [23].

Vineyards are a regionally dominant and intensively managed perennial crop type with frequent management interventions like ploughing and mowing of ground cover vegetation and inputs of large amounts of pesticides compared to other crops [24]. Wine growing landscapes often have a monocultural character due to the high profitability of the crop, but also because as a permanent crop type viticulture is excluded from the European Common Agricultural Policy’s requirements of designating ecological focus areas [25]. Nevertheless, vineyard areas have the potential to host high biodiversity, including rare and endangered species [26–28]. Still, only few bird species of wine growing areas breed directly in vineyards [29–32]. Most species depend on SNH types like hedges, woodland patches and grassland areas for nesting and food provisioning [31, 32]. These landscape elements have frequently been removed in the course of land consolidation schemes that aim at increasing field sizes or in order to maximise production [e.g. 33]. Another reason for the removal of woody landscape elements are fears that they might be reservoirs for grape pest species like spotted wing drosophila (*Drosophila suzukii*) [34, 35].

The Palatinate region in the south of the federal state of Rhineland-Palatinate is Germany’s largest wine-growing area. The study area is located west of the city of Landau along the edge of the Palatinate forest in the Upper Rhine Valley, where vineyards are the dominant land use. SNH structures are frequently located in riparian areas but hedges and other woody elements and open SNH types like grasslands and fallows can be found in the wider landscape as well. In order to find ways to restore vineyard bird diversity, it is crucial to gather information on the effects of landscape composition on birds at local and landscape scales. Therefore, we here aim to find out 1) how an increase in SNH cover affects breeding bird diversity and abundance in viticultural landscapes, 2) at which spatial scale breeding birds are most influenced by the presence of SNH, and 3) how the response to SNH cover differs between species, especially species of conservation concern, and different functional groups.

## Methods

### Study area

**Fig 1:**
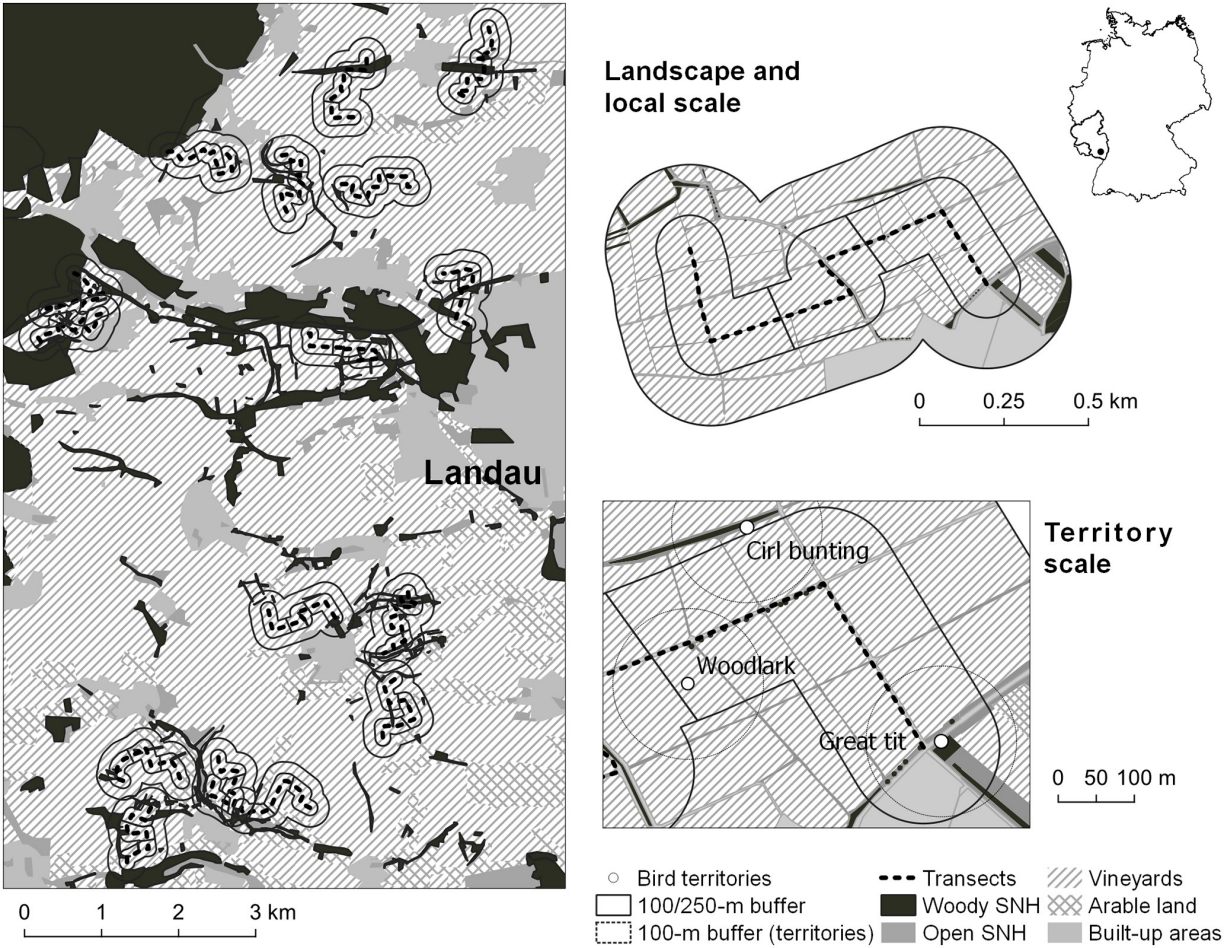
Map of the study area in the vineyards west of the city of Landau in the federal state of Rhineland Palatinate. The analyses were conducted on three different scales: on the scale of the entire landscapes (500 m radius), the transect scale (three transects per landscape with a buffer of 100 m radius) and the territory scale (100 m radius around territory centres).

The study was conducted in south-western Germany in the federal state of Rhineland-Palatinate. The Palatinate is situated at the western margin of the Upper Rhine Valley and is the largest wine-producing region in Germany. The mean annual rainfall is 675 mm (Landau), with warm summers (average temperature in July 18.8°C) and mild winters (average temperature in January 0.7°C, https://de.climate-data.org). The study sites were located in the vineyards west of the city of Landau (Fig 1). In the vineyards the ground vegetation is often managed in an alternating manner, where in spring every second inter-row is tilled while the next inter-row is covered with grassy vegetation that is used for vehicle access. Vineyards with completely bare ground or vegetation in all inter-rows are less common. Inter-rows are sometimes sown with annual ground cover mixtures after tillage in autumn or spring but are often bare during the first months of the year.

### Study design

Within the study area of approx. 50 km^2^ we chose 16 landscapes with a radius of 500 m (Fig 1) along a gradient in SNH cover (both woody and open SNH types). Land cover was classified in the following categories (ordered from high to low cover): vineyards, built-up areas (villages), arable land (mainly occurring in the eastern part of the study area), woody SNH types (woodland patches, hedges, tree rows, single trees and orchards) and open SNH types (meadows, pastures, grass strips, fallows and ruderal areas).

### Bird mapping

We conducted breeding bird surveys in spring 2019, following the standardised methodology described in Südbeck et al. (2005). Birds were mapped on a path that spanned from one side of each landscape to the other crossing its centre. Each path was split into three parts (transects) of approx. 500 m length each (Fig 1). Mapping took place in the early morning beginning at sunrise in good weather conditions (days without rain and wind), walking on vineyard paths. Only birds up to a distance of approx. 100 m to both sides of the transect were recorded. Each transect was mapped four times between March and June (mapping 1: 03/20-03/29, mapping 2: 04/15-04/23, mapping 3: 05/01-05/18, mapping 4: 05/23-06/02) [36]. After the fourth survey, for every species we determined the number and positions of territories according to the relevant time periods and indicative behaviour given in [36].

### Geographical analyses

Based on the results of the landscape mapping we calculated the cover of SNH (woody and open habitat types), vineyard cover and the cover of built-up and arable areas on three different scales (Fig 1): 1) on the scale of the entire landscapes (500 m radius, n = 16), 2) the transect scale (n = 48) and 3) the scale of individual territories (n = 300). For the transects, we placed a 100 m buffer around each one that was then intersected with the landscape in order to calculate the coverage of different habitat types within the buffer. Depending on their shape, transects comprised an average area of 9.6 ha (± 1.0 SD). At the territory scale, we placed 100 m buffers around each territory centre and intersected this buffer with the landscape as well to calculate the cover of different habitat types. The area a territory comprises is highly species specific but can also vary within species depending on the suitability of the habitat [31]. However, for most species found in this study, the area of approx. 3 ha within the buffer can be assumed to cover the territory as well as the surrounding habitat used for foraging and chick provisioning [31].

### Statistical analyses

Correlations between land cover types were tested using the functions *cor* and *cor*.*test* in R. At all scales (landscape, transect, territory) vineyard cover and the cover of SNH (sum of cover of open and woody SNH types) were negatively correlated. There was a positive correlation between the cover of open and woody SNH types (S1 Table).

In order to evaluate the influence of the surrounding landscape on breeding birds, we performed three types of analyses: 1) Analysis of bird species richness and number of territories, 2) compared the amount of SNH in the surroundings of each territory with the overall landscape mean for each bird species and 3) redundancy analysis to assess differences in community composition.

1. In order to analyse species richness and number of territories of birds with the cover of SNH as an explanatory variable, we fitted two types of models: a) linear models (*lm*) at the scale of the entire landscapes and b) mixed-effect models (*lmer*) at the transect scale. Due to the nestedness of the transects within landscapes, “landscape ID” was used as a random factor.
2. We then fitted null models to test whether the overall amount of SNH on the territory level differed from the landscape-wide average (i.e. the mean value of the 16 landscapes), both for all species taken together and for each species individually. Only species of which we had recorded at least five territories were tested individually. In these linear models the dependent variable was the difference between each territory’s cover of SNH and the landscape-wide average. The difference was regarded significant if the *P*-value of the intercept was <0.05.
3. Finally, the relationship between bird community composition at the transect scale and the cover of different habitat types (percentage of vineyards, arable land, built-up areas and SNH types, i.e. woodlots, hedges, tree rows and single trees, orchards and grasslands) as explanatory variables was assessed using partial redundancy analysis (RDA) with centred response data with the function *rda* from R package vegan [37]. Species that occurred on fewer than three transects were excluded from the data set prior to analysis. To assess statistical significance we used a permutation test with 9999 permutations with the function *permutest* from R package vegan [37].

All statistical analyses were conducted with R version 4.0.5 [38].

## Results

In total we recorded 300 territories of 33 breeding bird species. The most common species were great tit (*Parus major*, 56 territories), blackbird (*Turdus merula*, 39 terr.), Eurasian blackcap (*Sylvia atricapilla*, 31 terr.) and chiffchaff (*Phylloscopus collybita*, 16 terr.). Nine of the 33 species (55 terr.) are threatened or near threatened, i.e. they are either listed on the German Red List, the Red list for Rhineland-Palatinate or on both lists [10, 39]. Red-listed species included linnet (*Linaria cannabina*, 14 terr.), starling (*Sturnus vulgaris*, 11 terr.), cirl bunting (*Emberiza cirlus*, 12 terr.), yellowhammer (*Emberiza citrinella*, 7 terr.) and woodlark (*Lullula arborea*, 5 terr.). Some of the red-listed species found here are also part of the European farmland bird index (https://pecbms.info/trends-and-indicators/indicators) (Table 2).

**Fig 2:**
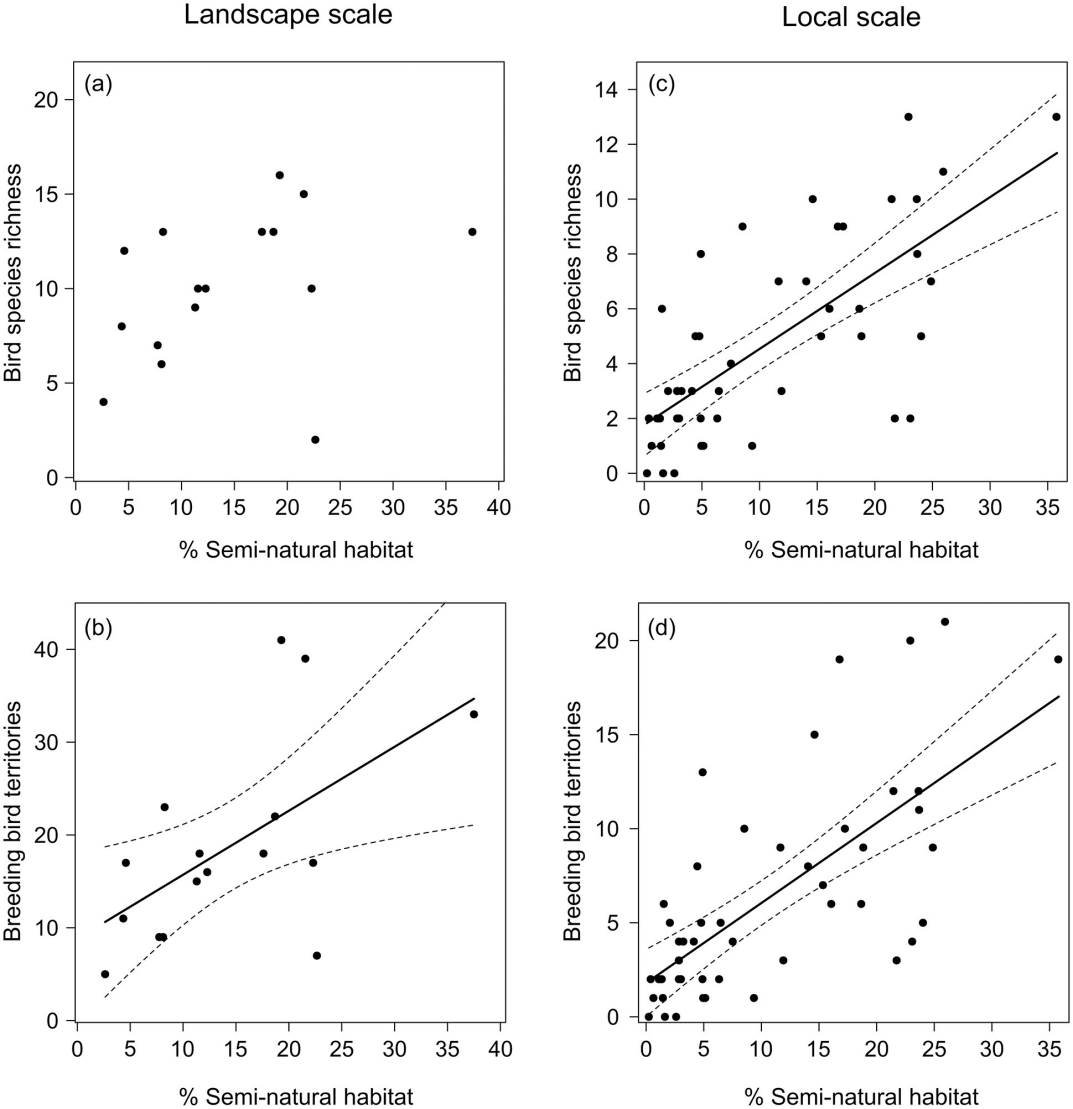
Response of bird species richness and number of breeding bird territories to an increasing proportion of SNH on the landscape scale (a+b, n = 16) and the transect scale (c+d, n = 48).

We found positive correlations of both bird species richness and the number of territories with the cover of SNH in the landscape. However, much stronger correlations were found at the transect than at the landscape scale (Fig 2, Table 1). At the transect scale, a 10% increase in SNH cover (representing on average 0.96 ha) added three bird species and 4.7 territories to the local community.

**Table 1:** Results of linear models (landscape scale) and linear mixed effects models (transect scale) on the effect of the cover of SNH (% SNH) on bird species richness and breeding bird territories.

			Estimate	SE	df	*t*	*P*
Landscape scale	Species richness	Intercept	8.01	1.92	14.00	4.17	**0.001**
		% SNH	0.15	0.12	14.00	1.24	0.236
	Territories	Intercept	8.50	4.56	14.00	1.86	0.084
		% SNH	0.76	0.29	14.00	2.60	**0.021**
			Estimate	SE	df	*t*	*P*
Transect scale	Species richness	Intercept	1.69	0.55	31.13	3.08	**0.004**
		% SNH	0.30	0.04	45.98	7.52	**<0.001**
	Territories	Intercept	1.58	0.84	30.77	1.88	0.070
		% SNH	0.47	0.06	45.50	7.44	**<0.001**

SE = standard error, df = degrees of freedom. *P*-values <0.05 are shown in bold.

**Fig 3:**
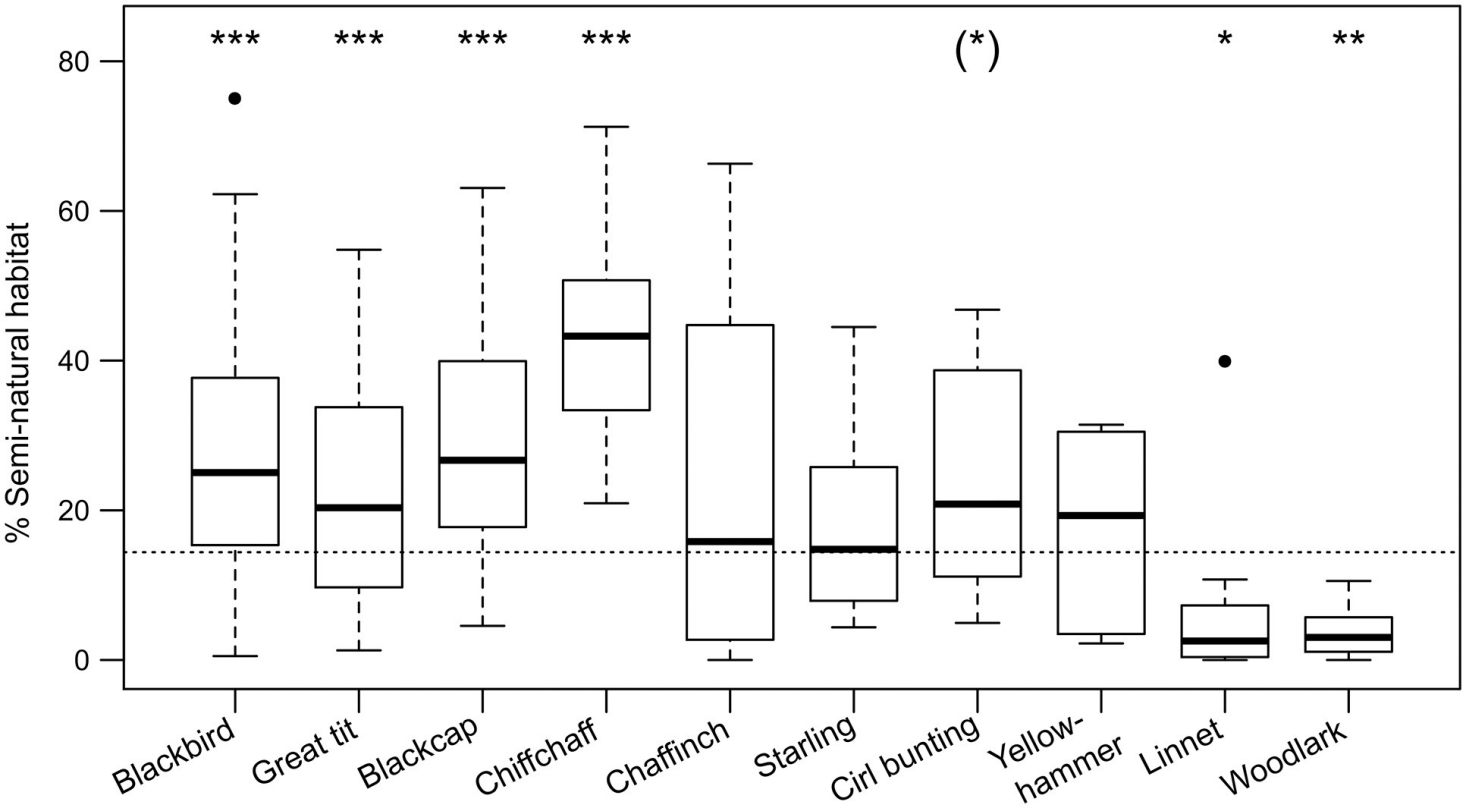
Cover of SNH in the territories of the five most common unthreatened breeding birds (left, blackbird to chaffinch) and the five most common red-listed breeding birds (right, starling to woodlark). The dashed line represents the overall cover of SNH averaged over the 16 landscapes (13.5%). *** *P* < 0.001, ** *P* < 0.01, * *P* < 0.05.

When comparing the cover of SNH at the territory level with the average cover across the 16 landscapes (13.5%), it became clear that the territories of most of the species (75%) and individuals (84%) contained more SNH than the landscape-wide average. By contrast, the territories of linnet, woodlark and white wagtail were almost completely dominated by vineyards (Fig 3, Table 2).

**Table 2:** Species recorded in the vineyards west of the city of Landau, the number of territories (Terr.) that were detected and whether these territories contained more or less than the landscape-wide average of SNH (13.5%, shown as a dashed line).

Species	*Scientific name*	Terr.	SNH (%) ± SD	Estimate	SE	t	*P*
Golden oriole	*Oriolus oriolus*	2	53.5 ± 5.1				
Short-toed treecreeper	*Certhia brachydacytyla*	2	50.4 ± 11.8				
Wren	*Troglodytes troglodytes*	3	45.3 ± 11.8				
Fieldfare	*Turdus pilaris*	2	43.5 ± 11.6				
Chiffchaff	*Phylloscopus collybita*	16	40.9 ± 10.3	27.4	2.6	10.6	**<0.001**
Barn swallow*	*Hirundo rustica*	1	37.3				
Blue tit	*Cyanistes caeruleus*	5	36.4 ± 9.5	22.9	4.3	5.4	**0.006**
Nightingale	*Luscinia megarhynchos*	5	36.1 ± 15.5	22.6	6.9	3.3	**0.031**
Robin	*Erithacus rubecula*	8	35.5 ± 16.7	21.9	5.9	3.7	**0.007**
Green woodpecker	*Picus viridis*	2	32.2 ± 14.1				
Common wood pigeon	*Columba palumbus*	5	30.0 ± 26.5	16.5	11.8	1.4	0.236
European stonechat	*Saxicola rubicola*	1	29.1				
Dunnock	*Prunella modularis*	2	28.3 ± 19.3				
Blackcap	*Sylvia atricapilla*	31	28.3 ± 14.3	14.8	2.6	5.8	**<0.001**
Blackbird	*Turdus merula*	39	27.0 ± 16.8	13.5	2.7	5.0	**<0.001**
Common redstart	*Phoenicurus phoenicurus*	1	23.6				
Great spotted woodp.	*Dendrocopos major*	2	23.3 ± 2.0				
Cirl bunting*	*Emberiza cirlus*	12	23.0 ± 14.3	9.5	4.1	2.3	**0.043**
Great tit	*Parus major*	56	22.8 ± 15.7	10.1	2.2	4.5	**<0.001**
Chaffinch	*Fringilla coelebs*	19	21.9 ± 20.5	8.4	4.7	1.8	0.093
House sparrow	*Passer domesticus*	4	20.8 ± 22.9				
Yellowhammer*	*Emberiza citrinella*	7	19.3 ± 12.8	5.7	4.8	1.2	0.280
Common whitethroat*	*Sylvia communis*	4	18.3 ± 8.6				
Starling*	*Sturnus vulgaris*	11	17.4 ± 11.5	3.9	3.5	1.1	0.292
Black redstart	*Phoenicurus ochrurus*	12	16.8 ± 18.4	3.3	5.3	0.6	0.546
Magpie	*Pica pica*	12	13.0 ± 13.6	-0.5	3.9	-0.1	0.892
Serin*	*Serinus serinus*	6	11.1 ± 8.1	-2.5	3.3	-0.7	0.490
Goldfinch	*Carduelis carduelis*	3	9.6 ± 5.2				
Greenfinch	*Carduelis chloris*	1	9.5				
Common linnet*	*Carduelis cannabina*	14	6.0 ± 10.5	-7.6	2.8	-2.7	**0.019**
Woodlark	*Lullula arborea*	5	4.0 ± 4.1	-9.5	1.8	-5.2	**0.006**
Carrion crow	*Corvus corone*	1	3.7				
White wagtail	*Motacilla alba*	6	3.2 ± 3.6	-10.3	1.5	-7.0	**0.001**
All species		300	23.8 ± 17.5	10.3	1.0	10.3	**<0.001**

The species are sorted from highest to lowest mean proportion of SNH (mean SNH %). Red-listed species are shaded in grey,

*marks species that are part of the European farmland bird index.

P-values <0.05 are shown in bold. SD = standard deviation, SE = standard error.

**Fig 4:**
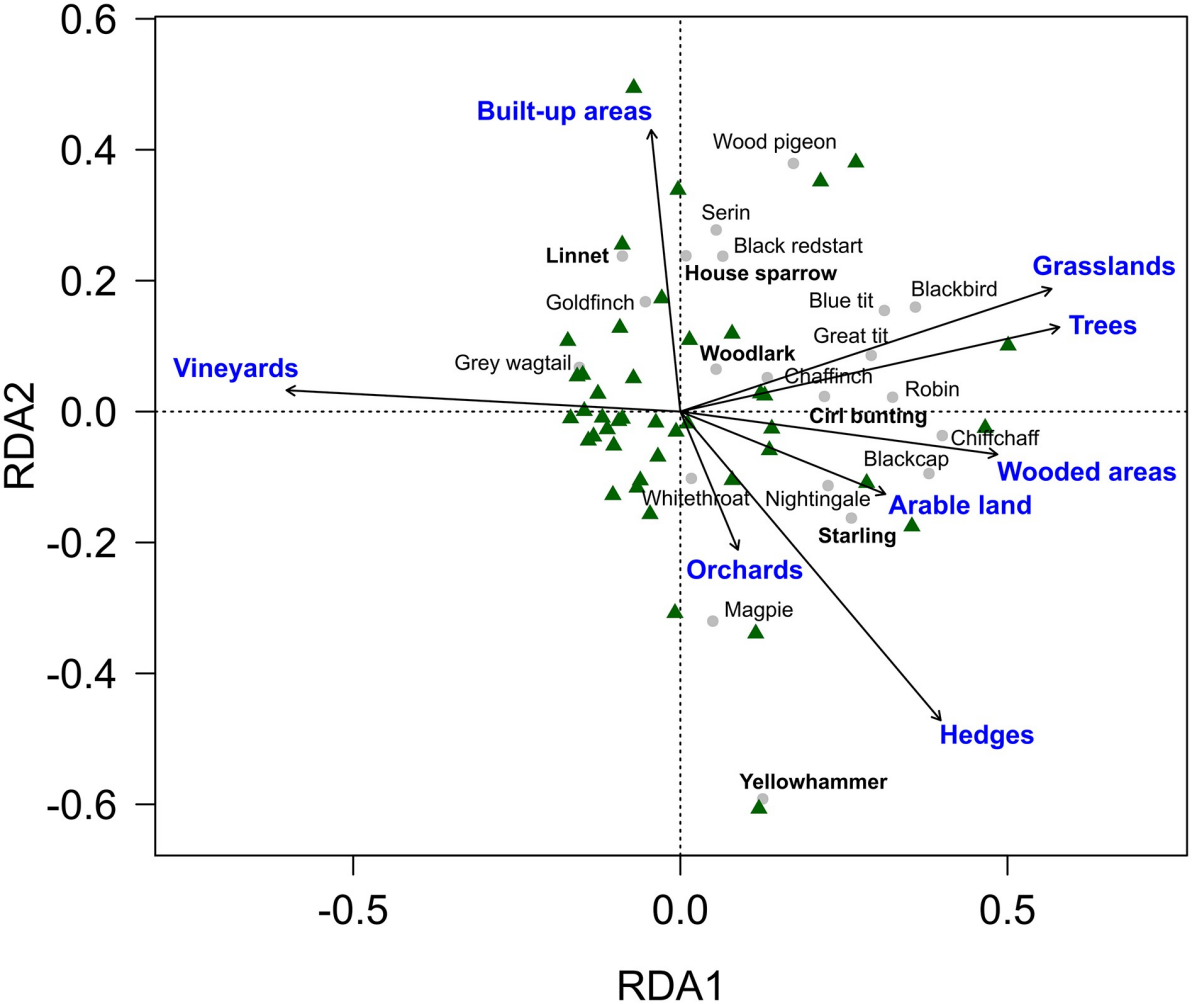
RDA plot showing differences in bird communities depending on the composition of the area around transects. Red-listed species are shown in bold characters.

The birds’ community composition was influenced by the composition of the surrounding landscape. All habitat types taken together explained 36% of the total compositional variation, while woody and open semi-natural habitat types explained 17% of the compositional variation. On the other hand, vineyard cover (on average 79% cover) had no significant effect on the birds’ community composition (Fig 4, Table 3). Most species were thus associated with SNH types or built-up areas while vineyards had hardly any species associated with them. Not even the woodlark, the only bird species that nests directly on the ground in vineyards in the study area, was associated with areas completely dominated by vineyards.

**Table 3:** Results of RDA analyses: Influence of vineyard cover, cover of arable land, built-up areas as well as different types of SNH on the community composition of birds on the transect scale.

Habitat type	Mean cover (%) ± SD	partial RDA % of variation	F	*P*
Vineyards	78.65 ± 15.49	2.20	1.34	0.183
Arable land	4.60 ± 10.15	2.48	1.51	0.114
Built-up areas	0.75 ± 2.30	2.66	1.62	0.077
Grassland	3.67 ± 3.51	2.25	1.39	0.164
Woodlots	2.47 ± 4.28	3.97	2.41	**0.011**
Hedges	1.33 ± 1.82	4.22	2.57	**0.004**
Trees	0.93 ± 1.50	4.05	2.46	**0.012**
Orchards	1.31 ± 2.83	2.83	1.72	0.065
Total	93.73 ± 2.60	36.41	2.79	**<0.001**

“Trees” comprises the cover of tree lines and single trees in the landscape. *P*-values <0.05 are shown in bold.

## Discussion

Territory numbers were largely proportional to the cover of SNH in the mapped area, showing a near complete dependence of breeding birds on SNH. In addition, the majority of species we recorded had a higher than average cover of SNH in the surroundings of their territory centre and community composition was influenced by the cover of SNH types but not by vineyard cover despite vineyards being the major land cover type in the study area. While positive effects of SNH on biodiversity are widely known from a range of organisms and farmland types [40, 41], most previous studies did not find such strong relationships [22, 23]. As sobering as this may appear regarding the low value of vineyards as habitat for the majority of bird species, our results point towards a straightforward strategy for bird conservation in viticultural landscapes: an increase in the cover of hedgerows, trees, woodland patches, extensively used orchards and permanent grassland. The comparison between the landscape and the transect scale emphasises that for birds despite being highly mobile, the local availability of nesting and nearby foraging structures is critical for the suitability of an area as breeding habitat. For the majority of the bird species recorded in this study, the breeding territories as well as the surrounding area used for foraging and chick provisioning only comprise a relatively small area (~1 to 5 ha) [31]. However, the analysis at territory level revealed important differences between species: while most species selected areas with higher amounts of SNH than the landscape-wide average for breeding, some species had an opposite preference, including linnet and woodlark which have high conservation value in viticultural landscapes.

The strong dependence of breeding birds on SNH has several explanations: Firstly, most of the species we recorded do not use vineyards as breeding habitat but require adjacent SNH structures like woodland patches and hedges for nesting [32]. They can be subdivided into three categories: cavity-nesting species (12 species), open-nesting species (15 species) and ground-nesting species (6 species) [32]. For cavity-nesting species, e.g. tits, starling and short-toed treecreeper, breeding in the wine canopy is precluded since cavities are lacking. Open nesting species like finches, blackcap, nightingale, linnet and dunnock require denser vegetation cover than the vine canopy can offer, especially in spring at the beginning of the breeding season. Despite being a perennial crop with a permanent cover of woody vegetation, vineyards tend to be subject to frequent management interventions including pesticide spraying and leaf removal during the growing season. Therefore, in many cases nests built within the wine canopy are abandoned [42]. New wine pruning techniques like minimal pruning that lead to a denser wine canopy were shown to increase the number of nests, but breeding success was equally low [42]. Finally, species like leaf warblers (*Phylloscopus sp*.), cirl bunting, robin and yellowhammer breed on or close to the ground but require dense vegetation cover to hide their nest [32], making vineyards with their short swards in the inter-rows and frequent passages of farming vehicles between vines unsuitable for breeding.

Therefore, in order to increase nest site availability, off-field agri-environmental schemes, e.g. maintaining and replanting hedges and trees and the maintenance of extensively used grassy vegetation areas are likely to be the most effective and straightforward way to enhance bird diversity and abundance in our study area and other similarly structured wine-growing landscapes [21]. For the woodlark, the only ground nesting species that breeds directly in vineyards in the study area [43, 44], ground cultivation management like mowing and tilling of inter-rows is likely to be a limiting factor as well [43]. Could show that areas dominated by forbs and with short vegetation are more likely to be selected as territories. Alternative sward management regimes like the establishment of permanent low-growing swards or sheep grazing with small sheep breeds that can only reach the lowest leaves of the canopy [45] could thus be options to consider. The removal of the lower leaves by sheep, which would otherwise shade the grapes is a desired effect that increases grape quality and is conventionally done by hand or with specialised machinery [45].

A second explanation of the strong dependence of birds on SNH could be the entailed availability of food resources in the close surroundings of the birds’ nest sites. Feeding areas with ample food supplies near the nest reduce the parent birds’ travel costs in terms of both energy and time [46, 47] and thus make it easier for them to sufficiently feed their young which is key for their growth and survival [48]. During the breeding season, the majority of the species we recorded (84%) partly or fully depend on invertebrates in order to raise their chicks [32]. SNH elements like hedges or woodland patches are structurally complex and thus offer ideal feeding conditions for leaf-gleaning species like tits, typical warblers (*Sylvia sp*.) and leaf warblers [49]. Especially early in the season the vine canopy is structurally much less complex since, in order to harvest good quality grapes, vines must be pruned every year and most of the previous year’s growth is removed. Many arthropods in hedges are predators that may help prevent outbreaks of insect pests within vineyards [50] and it has been shown that spiders colonise vineyards from adjacent hedges [51]. Pest control could be enhanced through a denser network of SNH elements since the occurrence of natural enemies within vineyards has been shown to be negatively correlated with the distance from the nearest hedge [52]. Although hedgerows are seen as potential reservoirs for fruit flies (e.g. *Drosophila suzukii*), it has been shown that hedgerows are not linked with grape infestation rates in adjacent vineyards [35]. Furthermore, arthropod consumption and thus pest control by birds could be compromised through the low number of bird territories in landscapes dominated by vineyards [49]. The threat of fungal infections often requires ten or more fungicide applications per season to maintain high yields as well as wine quality [26, 53]. Although targeted at fungi, fungicide applications can negatively affect arthropods [54]. Here, the gradual conversion towards the cultivation of novel fungus resistant grape varieties might be a promising approach. For example, vineyards with a reduced number of pesticide applications in concordance with a minimal pruning approach have been shown to host more predatory mites [55]. Vineyards with a minimal pruning regime might also be more attractive for birds since the vine canopy is relatively dense all year round, and offers a greater structural diversity for arthropods [54].

For the large group of bird species that mostly forage on the ground, including buntings, finches, redstarts, woodlark and starling, grassland areas are an important feeding habitat [32]. If arthropods are available, the inter-rows of vineyards in the surroundings of their nest site can be attractive for ground-foraging species like finches, linnet, robin and buntings [56, 57]. Vineyards with a diverse flower-rich ground cover can furthermore positively affect other taxa like bees [27] and spiders [26]. Therefore, as mentioned above, grazing with sheep could be an alternative to create structurally complex swards that support diverse arthropod communities, also linked with the sheep’s dung [45].

## Conclusions

According to our results, the planting of hedges, trees and woodland patches and the maintenance of extensively used orchards and grassland areas is a straightforward way to reverse recent declines of birds in vineyard areas. Benefits of an increase in SNH cover would reach beyond bird conservation. It might help reduce erosion that can be a problem especially on steep slopes, increase pest control through natural enemies that spill over to cultivated areas and also positively affect other taxa that like birds depend on SNH.

Landscape planning should take on a differentiated approach that creates a landscape mosaic in which heterogeneity is key. To foster all birds that occur, areas dominated by vineyards and grassland areas for open farmland species need to alternate with more heterogeneous areas for typical birds of hedges and woodland. Greening schemes should include all structures that these species require for successful territory establishment, breeding and chick raising, i.e. dense vegetation like blackberry hedges for nesting, single trees as songposts and diverse grassland areas for feeding.

## Supporting information

S1 Table Correlations between the different land cover types. Correlations with a *P*-value <0.05 are shown in bold.
(DOCX)
