# Supplementary material for: Increase in breeding bird abundance and diversity with semi-natural habitat in vineyard landscapes
Source: PLoS One. 2023 Aug 21;18(8):e0284254. doi: 10.1371/journal.pone.0284254 (PMC10441799; doi:10.1371/journal.pone.0284254)
Supplement: S1 Table — Correlations with a P-value <0.05 are shown in bold. (DOCX) [file pone.0284254.s001.docx]

**Supporting information**

|  |  | | Open SNH | | Woody SNH | | Vineyards | | Arable land | |
| --- | --- | --- | --- | --- | --- | --- | --- | --- | --- | --- |
| Landscape scale | Woody SNH | | **0.55** | |  | |  | |  | |
|  | Vineyards | | **-0.76** | | **-0.66** | |  | |  | |
|  | Arable land | | 0.39 | | -0.09 | | **-0.54** | |  | |
|  | Built-up areas | | -0.03 | | -0.08 | | -0.14 | | -0.34 | |
|  |  | |  | |  | |  | |  | |
| Transect scale | Woody SNH | | **0.52** | |  | |  | |  | |
|  | Vineyards | | **-0.63** | | **-0.75** | |  | |  | |
|  | Arable land | | 0.16 | | 0.26 | | **-0.73** | |  | |
|  | Built-up areas | | 0.03 | | -0.11 | | -0.11 | | -0.08 | |
|  |  | |  | |  | |  | |  | |
| Territory scale | Woody SNH | | **0.40** | |  | |  | |  | |
|  | Vineyards | | **-0.48** | | **-0.60** | |  | |  | |
|  | Arable land | | 0.04 | | 0.27 | | **-0.64** | |  | |
|  | Built-up areas | | 0.08 | | **-0.08** | | **-0.26** | | -0.10 | |
|  |  |  | |  | |  | |  | |  |

**Table S1.** Correlations between the different land cover types. Correlations with a *P*-value <0.05 are shown in bold.
